# Cognitive Workload of Tugboat Captains in Realistic Scenarios: Adaptive Spatial Filtering for Transfer Between Conditions

**DOI:** 10.3389/fnhum.2022.818770

**Published:** 2022-01-27

**Authors:** Daniel Miklody, Benjamin Blankertz

**Affiliations:** Electrical Engineering and Computer Science Department, Neurotechnology Group, Institute of Software Engineering and Theoretical Computer Science, Technische Universität Berlin, Berlin, Germany

**Keywords:** cognitive workload, EEG, spatial filtering, beamforming, non-stationarity, adaptation

## Abstract

Changing and often class-dependent non-stationarities of signals are a big challenge in the transfer of common findings in cognitive workload estimation using Electroencephalography (EEG) from laboratory experiments to realistic scenarios or other experiments. Additionally, it often remains an open question whether actual cognitive workload reflected by brain signals was the main contribution to the estimation or discriminative and class-dependent muscle and eye activity, which can be secondary effects of changing workload levels. Within this study, we investigated a novel approach to spatial filtering based on beamforming adapted to changing settings. We compare it to no spatial filtering and Common Spatial Patterns (CSP). We used a realistic maneuvering task, as well as an auditory *n-back* secondary task on a tugboat simulator as two different conditions to induce workload changes on professional tugboat captains. Apart from the typical within condition classification, we investigated the ability of the different classification methods to transfer between the *n-back* condition and the maneuvering task. The results show a clear advantage of the proposed approach over the others in the challenging transfer setting. While no filtering leads to lowest within-condition normalized classification loss on average in two scenarios (22 and 10%), our approach using adaptive beamforming (30 and 18%) performs comparably to CSP (33 and 15%). Importantly, in the transfer from one to another setting, no filtering and CSP lead to performance around chance level (45 to 53%), while our approach in contrast is the only one capable of classifying in all other scenarios (34 and 35%) with a significant difference from chance level. The changing signal composition over the scenarios leads to a need to adapt the spatial filtering in order to be transferable. With our approach, the transfer is successful due to filtering being optimized for the extraction of neural components and additional investigation of their scalp patterns revealed mainly neural origin. Interesting findings are that rather the patterns slightly change between conditions. We conclude that the approaches with low normalized loss depend on eye and muscle activity which is successful for classification within conditions, but fail in the classifier transfer since eye and muscle contributions are highly condition-specific.

## 1. Introduction

The estimation of cognitive workload (Gevins et al., 1995; Pope et al., 1995; Gevins and Smith, 2003) delivers various application scenario that have been and are heavily researched. In particular in stressful working environments, such as air-traffic control (Brookings et al., 1996; Aricò et al., 2016; Borghini et al., 2020), pilots and car drivers (Kohlmorgen et al., 2007; Borghini et al., 2014), as well as ship captains (Miklody et al., 2016) and other applications (Naumann et al., 2016; Kosti et al., 2018; Tremmel et al., 2019), the assessment of current individual workload level can help to detect dangerous situations, as well as in training, interface design and the design of infrastructure, such as airports, harbors, and bridges. In realistic situations, individual *post-hoc* questionnaires and qualitative observations often remain the tool of choice, as other methods such as artificial secondary tasks affect the actual working situation and most bio-physiological measurements are still impractical and unreliable at the same time.

However, questionnaires and qualitative observations often remain rather subjective and discrete in time, while a continuous and objective estimation of current cognitive workload is desirable. Thus, secondary tasks are often used, which a participant has to solve while performing the actual task. The current error rate in the secondary can then, e.g., be used to indirectly estimate the actual workload of the primary task. This distracts the participants on the one hand from the main task and remains indirect on the other. Thus, bio-physiological measurements using, e.g., skin resistance (SR), heart rate (HR), eye tracking or EEG are expected to be less intrusive while delivering real-time objective estimation of cognitive workload. Except for eye tracking, most methods work in experimental laboratory settings, while the transfer to other experimental or more realistic settings mostly fails. Eye tracking in contrast has reached a higher level of relevance through higher reliability combined with ease of setup and is thus used, e.g., in drowsiness detection systems in cars, etc.

In particular, the electrophysiological measurements like skin resistance, electrocardiography (ECG) for heart rate and EEG suffer from more intrusive technical details and a low signal-to-noise ratio (SNR). Additionally, the signal components mostly considered as noise or artifacts stemming from eye and body movements are highly situation and individual subject (strategy) dependent, rendering the development of a general workload estimator challenging. However, for the estimation of *cognitive* workload, EEG is one of the few widely applicable and non-invasive system with direct access to neural activity. All other mentioned methods measure the effect of cognitive workload on the physiological stress level (SR, ECG, pupillometry through eye tracking) or behavior (eyelid closure, etc.)

Cognitive workload is reflected in different components of brain activity. Considering the present target application, modulations of event-related potentials due to workload (Polich, 1987; Kramer, 1991; Kok, 1997) are not relevant, since there are no controlled and continuously repeated stimuli. Therefore, we concentrate on workload-induced modulations of spontaneous brain activity.

The power of oscillatory brain activity in the theta frequency range (4 to 7 Hz) in frontal brain regions have been found to positively correlate with the level of workload, see, e.g., (Gevins et al., 1998; Smith et al., 2001; Holm et al., 2009).

Regarding the more prominent alpha frequency band, most studies report a negative correlation of cognitive workload and alpha power at parito-occipital scalp locations, see, e.g., (Gevins and Smith, 2003; Holm et al., 2009). However, these studies used tasks in the visual modality to induce workload, such that one can only derive the implication of alpha reduction for workload in visual resources. In general, the functional role of alpha band oscillations is not yet conclusive. For a memory task in the auditory domain, Gundel and Wilson (1992) reports a modulation of theta oscillations only, but no modulations of the alpha rhythm. Some studies using auditory stimulation even found an increase in alpha activity with increasing workload (Legewie et al., 1969; Galin et al., 1978; Markand, 1990; Kohlmorgen et al., 2007). A possible interpretation is provided by the hypothesis of functional inhibition, which postulates that strong alpha activity reflects active inhibition of task-irrelevant processes (Klimesch et al., 1999): when the critical processing load is in the non-visual, the visual areas are actively deactivated.

In a series of experiments, we found out, that the lowest classification errors were mostly reached for uncleaned (and thus artifact polluted) data while the transfer between different settings did not work (Miklody et al., 2016). This lead to further investigations, leaving a hint toward actually discriminative artifacts within conditions that make a transfer from one to another setting with different behavioral settings challenging.

In this study, we investigate a novel approach that used the information from a head model to extract neural information only. We employ beamformers suppressing other sources measured in the data and use adaptation to account for changing environments and tasks in different experimental conditions.

## 2. Materials and Methods

The data used within this study stems from a study published in Miklody et al. (2016) and is part of a realistic cognitive workload experiment involving professional ship captains. The experiment consisted of two conditions where the workload was manipulated by the difficulty of the maneuvering task in one and by a secondary *n-back* task (Kirchner, 1958) in the other condition. The classification followed classical approaches for spatial filtering of continuous EEG data. After band-pass filtering separately to alpha and theta band, the data was spatially filtered followed by calculation of the logarithm of variances for feature linearization. This feature vector was then fed into a Linear Discriminant Analysis (LDA) classifier that was trained on high vs. low workload discrimination.

### 2.1. Participants

10 professional tugboat captains (all male, age 30–65 years) working in the port of Rotterdam were acquired for an extensive session of experiments in a professional ship simulator. They collaborated voluntarily while being financially compensated for their participation. Subject 8 had to be excluded from analysis as he got seasick during the first run and could not continue the experiments. The study was approved by the committee of the ethical department of Philips, the Netherlands, as we collaborated with them on this project. An informed written consent was obtained from all participants.

### 2.2. Experimental Setup

The EEG was recorded with BrainAmps from BrainProducts on a sampling rate of 1 kHz with 64 channels (except for subject 2 with 32 electrodes due to an instrumentation error). The Ag/AgCl-gel electrodes were placed in a standard 10/10 system and impedance was reduced to below 5 kOhms before the experiments and checked between sessions. The data was recorded with a linked mastoid reference behind both ears. The simulator was a professional ship simulator bridge optimized for tugboat missions which can be observed in Figure 2. It consisted of a 360° projected screen around a set of ordinary tugboat controls. This included several additional screens for radar and ship-parameters. It was built and is operated for realistic training of professional captains by MARIN.

### 2.3. Experimental Paradigm

The experiment consisted of three phases of around 40 min duration. Phase 1+3 were identical tasks with cognitive workload alternated by the difficulty of the maneuvering task while phase 2 was low workload sailing with an auditory *n-back* secondary task to induce two level of workload.

#### 2.3.1. Maneuver

In the maneuver condition, cognitive workload was induced by the changing difficulty of the maneuvering task itself. The tug-boats had to connect to a large container-ship in the port of Rotterdam and tow sailing backwards in a so-called *bow-to-bow* setting. Additionally, the weather and sea conditions were changed to difficult for the high workload level. The low workload task was following the container-ship astern with calm sea and clear visibility. The low workload blocks were 5 min at the beginning of each run and 10 min of connecting and pulling after moving to the bow of the container ship were used as high workload tasks. This was repeated once, leading to a duration of ~40 min.

#### 2.3.2. *n-back*

The sailing task during the *n-back* condition was the same as the low workload in the maneuver condition—a constant following of the container ship. The *n-back* (Kirchner, 1958) was added as a secondary task, with auditory playback of numbers 0–9. The participant had to push a button for matching *n-back* numbers. The low condition was a 0-back and the high condition a 2-back. In the *n-back*, 4 min blocks of high/low workload were constantly alternated for 5 repetitions of each leading to 10 runs within 40 min.

### 2.4. Preprocessing

As reviewed in the introduction, changes in mental workload were found to be linked with the modulation of α (8–13Hz) and θ (4–7Hz) band activity. While a change in α activity is mostly related to attention and/or idling in the corresponding cortices, frontal θ activity seems to be related to working memory. Additionally, these two bands are least affected by eye and muscle contributions. Therefore, we have limited our analysis to two band-pass filtered signals for each channel in these frequency bands. The raw signal was filtered with digital butterworth-type band-pass filters of order 4 for each of the two bands (α 8–13Hz and θ 4–7Hz).

After this, the data was cut into non-overlapping epochs of 60 s, which in a previous study on this data was found to be the most stable length with the lowest errors in cognitive workload classification (Miklody et al., 2016).

### 2.5. General Linear Model

Based on quasi-electrostatic assumptions for EEG (Sarvas, 1987), the connection between source activities *s*(*t*) and the measured signal *x*(*t*) is a linear combination of all sources. The common model for EEG is thus assumed to be the mixture of sources described by a mixing matrix *A* in combination with some often undefined noise term η:


(1) $$\begin{array}{l} x=As+\eta \end{array}$$

The noise term is generally a mixture of all signal contributors that are not taken care of as sources within the model. If we now look at continuous oscillatory activity within the EEG, we are usually interested in the variances or covariances of the *sources*. We can only measure the covariance of the *data x*, but we can use this to estimate the activity of the underlying sources. If we take Equation (1) and apply it to the covariance matrices, we get:


(2) $$\begin{array}{l} \Sigma_{x}=A\Sigma_{s}A^{⊺}+\Sigma_{\eta } \end{array}$$

### 2.6. Spatial Filtering

We can estimate the source activity *s* from *x* using an array of spatial filters *W*:


(3) $$\begin{array}{l} ŝ=W^{⊺}x \end{array}$$

or in covariance matrices:


(4) $$\begin{array}{l} \Sigma_{ŝ}=W^{⊺}\Sigma_{x}W \end{array}$$

These filters are spatial because the multi-variate time-series *x*(*t*) consists of location dependent variables.

Combining Equations (1) and (4) implies that the spatial filters in general, should also take care of eliminating the influence of the noise:


$$\begin{array}{l} \Sigma_{ŝ}=W^{⊺}\Sigma_{x}W=W^{⊺}\left(A\Sigma_{s}A^{⊺}+\Sigma_{\eta }\right)\;W \end{array}$$

This is the reason, why in general the spatial filters are not just the inverse or pseudo-inverse of the patterns matrix *A*, as they also (have to) depend on the noise in order to extract only the source activity *s* (compare, e.g., Haufe et al., 2014). This has also some implications on changing noise and signal statistics, e.g., for different scenarios.

Mostly, also muscle and eye contributors (or artifacts) are not directly modeled as sources but considered noise, so a changing contribution of these also implies a need to adopt the filters. Additionally, depending on contact impedances of electrodes the instrumentation noise can change, as well as the signal levels of the neural sources. Nevertheless, also the neural sources themselves are known to be non-stationary (Linkenkaer-Hansen et al., 2001), which renders the transfer of spatial filters from one experiment to another challenging.

If additionally the experimental paradigm changes, parts of the filter might be over-fitted toward one special type of setting, which poses the need to build filters, that extract meaningful and general activity that is common in different environments.

The corresponding patterns to the filters are calculated by (compare Haufe et al., 2014):


(5) $$\begin{array}{l} A=\Sigma_{x}W\Sigma_{s}^{-1} \end{array}$$

We used either no filtering, Common Spatial Patterns (CSP) or our own beamforming (BF) approach on the data.

#### 2.6.1. Common Spatial Patterns

Common Spatial Patterns (CSP) is a data-driven spatial filter that optimizes to separate the data in covariances between two conditions or classes of data (Fukunaga, 1990; Koles, 1991; Ramoser et al., 2000; Blanchard and Blankertz, 2004). It is among the most-effective tools used in Brain-Computer-Interfacing, frequently used in the classification of motor imagery data (Blankertz et al., 2008) within experimental paradigms. It is however prone to issues of over-fitting, in particular to singular muscle and eye movements (Blankertz et al., 2008) but also to anything that differs in the statistics between the conditions, as well as spurious correlations.

The set of filters can be defined in several similar ways, with the objective that the spatially filtered signals maximize the variances for one class while minimizing it for the other class. The mathematical optimization for a 2-class problem can be formulated as:


(6) $$\begin{array}{l} w^{*}=arg\;max_{w}\frac{w^{⊺}\Sigma_{1}w}{w^{⊺}\left(\Sigma_{1}+\Sigma_{2}\right)\;w} \end{array}$$

where w^*^ is the optimal filter being the *argmax* of a projection using *w* of one class covariance matrix Σ_1_ in the nominator and a sum of the covariances of both classes in the denominator. (*Note: This is equivalent to other formulations using only* Σ_2_ *or the difference* Σ_1_ − Σ_2_ *in the denominator but leads to different eigenvalues*). The optimization Equation (6) is solved by generalized eigenvalue decomposition, which does not only provide one solution vector w^*^, but a matrix of Eigenvectors *W*—which are spatial filters—as its columns.

Usually, a certain number of Eigenvectors is selected depending on the Eigenvalues. Since spatial filters maximizing and minimizing the objective function (Rayleigh quotient) are both equally useful, Eigenvalues from both ends of the Eigenvalue spectrum are selected. According to the literature, no more than three plus three filters are needed in most EEG data sets. This was also the strategy used in this paper.

#### 2.6.2. Beamforming

Beamforming is an approach that is widely used in EEG to reconstruct source activity from a Region Of Interest (ROI) by employing the assumed mixing properties of the signal in combination with measured signal statistics. It has the advantage over CSP of delivering more generic components independent of the current class differences, but only based on the forward model of a certain area and the measured sum of source activity and noise. It can thus be optimized to filter out the activity of a certain area for a certain noise. In some experiments, it has been shown to obtain a comparable performance to CSP (Grosse-Wentrup et al., 2009). We use it here for continuously adapting to potential changes in the components themselves, noise, background or other contributors' activity.

In an approach similar to Grosse-Wentrup et al. (2009), we use the optimization


(7) $$\begin{array}{l} w^{*}=arg\;max_{w}\frac{w^{⊺}\Sigma_{ROI}w}{w^{⊺}\Sigma_{x}w} \end{array}$$

with


(8) $$\begin{array}{l} \Sigma_{ROI}=A_{ROI}^{⊺}\Sigma_{s}A_{ROI} \end{array}$$

and signal covariance matrix Σ_*x*_. As for CSP, filters are obtained by generalized eigenvalue decomposition. However, here Eigenvectors that maximize the objective function do not necessarily have a meaning for discriminating meaningful components like with CSP. Hence, all components were used.

In general, the beamformers are used for reconstructing the activity that comes from a certain region. For motor imagery BCIs they have been used for the left and right motor cortices for example and the results compare to CSP in performance. As we realized in our previous study that in a complex scenario and with changing tasks from auditory to visual we cannot find clear centers of activity neither for the alpha nor for the theta band, we decided on using the whole brain as a single ROI and let the algorithm find the centers automatically that are discriminable well from background activity. An ROI is generally defined by a certain number of sources in vicinity to some cortex area, and then the algorithm finds the mean signal from that area that is well discriminable. Setting the whole brain as an ROI potentially leads to unspecific patterns as combinations of many sources can reproduce any arbitrary pattern, but the result are well adaptable and separate filters that maximize correlated activity over noise.

#### 2.6.3. Adaptation of Spatial Filters

Adaptation can be reached on several levels: the classifier and the spatial filters can be adapted. Here, we only investigate the adaptation of spatial filters. In Grosse-Wentrup et al. (2009), the beamformers were estimated block-wise for each epoch, which introduces a less stable estimation. We decided for another approach using slower adaptation. The covariance matrix of the data Σ_*x*_ can be continuously estimated, and this can serve as a basis for adaptation of CSP and the beamformer. Note, that this is an unsupervised adaptation.

For a sequence of windows of samples (or epochs) *x*_*i*_, we define for *M* > 0 the covariance matrix $\Sigma_{x}^{\left(M\right)}$ which is updated with windows up to *x*_*M*_ by


(9) $$\begin{array}{l} \Sigma_{x}^{\left(M\right)}=\left(1-\lambda \right)\Sigma_{x}^{\left(M-1\right)}+\lambda \Sigma_{x_{M}} \end{array}$$

where $\Sigma_{x}^{\left(0\right)}$ is an initial covariance matrix, e.g., estimated from training data.

#### 2.6.4. Overview Over Implemented Methods

- *None*: No spatial filtering was used.
- *CSP*: CSP was trained on one condition and directly applied to the other.
- *CSPa*: CSP was trained on one condition and continuously updated in an unsupervised fashion by recalculating it with a new Σ_1_ + Σ_2_ in the denominator.
- *BF*: CSP was trained on one condition and directly applied to the other.
- *BFa*: BF was trained on one condition and continuously updated in an unsupervised fashion by recalculating it with a new Σ_*x*_ in the denominator.

### 2.7. Feature Extraction and Classification

For classification, we used a linear classifier applied to the vector of the logarithm of the variances of spatially filtered data. This is a common approach in classifying continuous oscillatory EEG data. The logarithm is applied to transform the variances which are χ^2^ distributed, to achieve distributions that are well separable using Linear Discriminant Analysis (LDA) (Fisher, 1936), i.e., Gaussian distributions (with equal covariance matrices in both classes). Alternatively, one can use a non-linear classifier that is derived as the optimal classifier for χ^2^ distributions. While this approach is theoretically appealing, it was found to result in higher error rates on real-world data because it is highly dependent on the stationarity of the data (Miklody, 2020).

### 2.8. Validation

In order to properly estimate the validity of the data, several special measures have to be taken into account for EEG data. As the data is highly auto-correlated and so not i.i.d., random sampling for cross-validation leads to information-leakage between test and training data. Therefore, chronological block-wise sampling is done for a leave-one-block-out cross-validation within conditions (Lemm et al., 2011). This problem does not apply for the transfer of filters and classifiers from one condition to another, as the data originates from separated time intervals and even another setting.

Additionally, it is essential that spatial filters are optimized on training data only. This is particularly important for CSP, since it employs label information (Blankertz et al., 2008). The block-wise sampling lead to 10-fold cross-validation in the *n-back* and 2-fold in each of the *maneuver* condition. Together with the 1-min time-windows this lead to 72 observations in training vs. 8 test for *n-back* and 20 test/10 training for *maneuver*. Between conditions refers to an external validation where a direct measurement of all statistics is applicable without any additional sampling. This lead to 80 observations for testing or training on the *n-back* condition and 30 for the *maneuver*.

### 2.9. Statistical Measures

All classification results in the this study are given by a class-wise normalized loss which delivers the advantage of having one clear score per result but incorporating the loss in both classes. The average per subject losses were tested using a Wilcoxon signed rank test to see whether they deviate significantly from chance level (50% for a two-class problem).

## 3. Results

### 3.1. Classification

**Figure 1:**
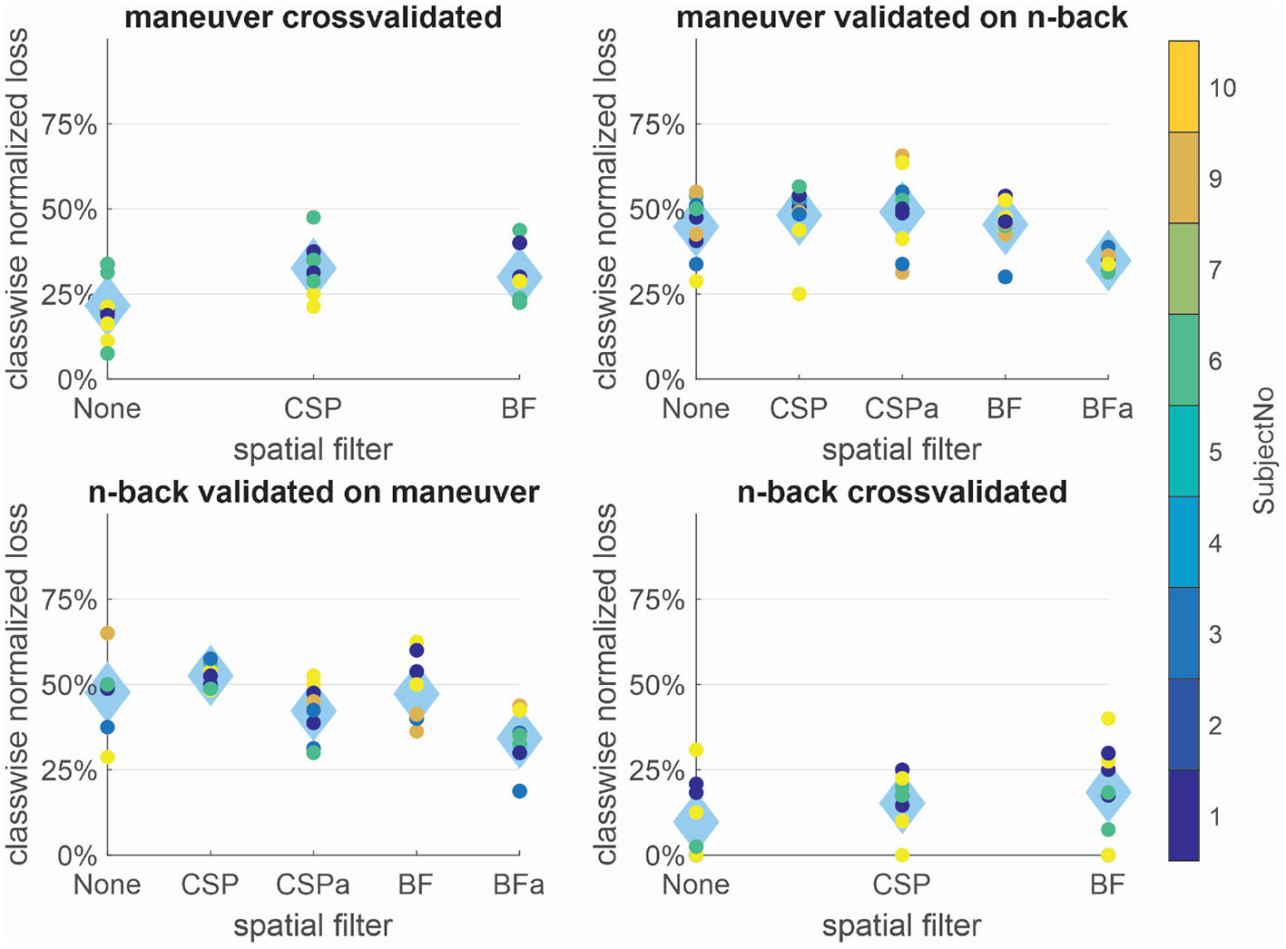
Within and between condition class-wise normalized losses for different spatial filtering approaches: Within conditions no spatial filtering leads to the lowest errors, while for the transfer between conditions, the non-data-dependent patterns of beamforming in combination with a constant adaptation is the only reliable approach. Dots show results for single subjects, while diamonds depict the average over subjects.

While the normalized losses were only very low within the *n-back* condition, the transfer between conditions was possible with the adapting BF approach in both directions. This is remarkably regarding the very different tasks. For joining the results of the maneuvering phases 1+3, the average normalized loss was used, while preceding training and evaluation remained separately. In Figure 1, we can see the normalized classification loss cross-validated within sessions and for the transfer of the processing and classification from one condition to another.

#### 3.1.1. Within Condition

Within condition, no spatial filtering lead to the lowest average normalized loss in both conditions—*n-back* (10%) and maneuver (22%). BF and CSP worked comparably within conditions, while for the *n-back*, CSP seemed to have a slight advantage (15%) over BF (18%). In the maneuvering condition normalized losses were, in general, higher for all approaches (33% for CSP, 30% for BF). All within condition results were significantly (α < 0.005) lower than chance level (50%).

#### 3.1.2. Transfer Between Conditions

The experimental paradigms differ strongly, and the transfer is around chance level without spatial filter adaptation for all approaches. Trained on the more controlled *n-back* condition and applied to the maneuvering, CSP and BF with adaptation classification results only differ significantly (CSP α < 0.05 and BF α < 0.005) from chance level, while BF leads to lower average errors (34%) than CSP (42%). The pipeline trained on the maneuvering task seems to generalize less well, as the normalized losses are around chance level (no filtering and BF without adaptation both 45%) except for BF with adaptation (35%, α < 0.005).

### 3.2. Scalp Patterns

Scalp patterns have been calculated from the spatial filters by Equation 5. Investigating them revealed where the components might have come from. We investigated the most discriminative patterns by cross-validation results for single component classification in order to get insights onto which components were important in which condition. For the patterns of BF with adaptation, the filters at the end of the constant update are displayed.

**Figure 2:**
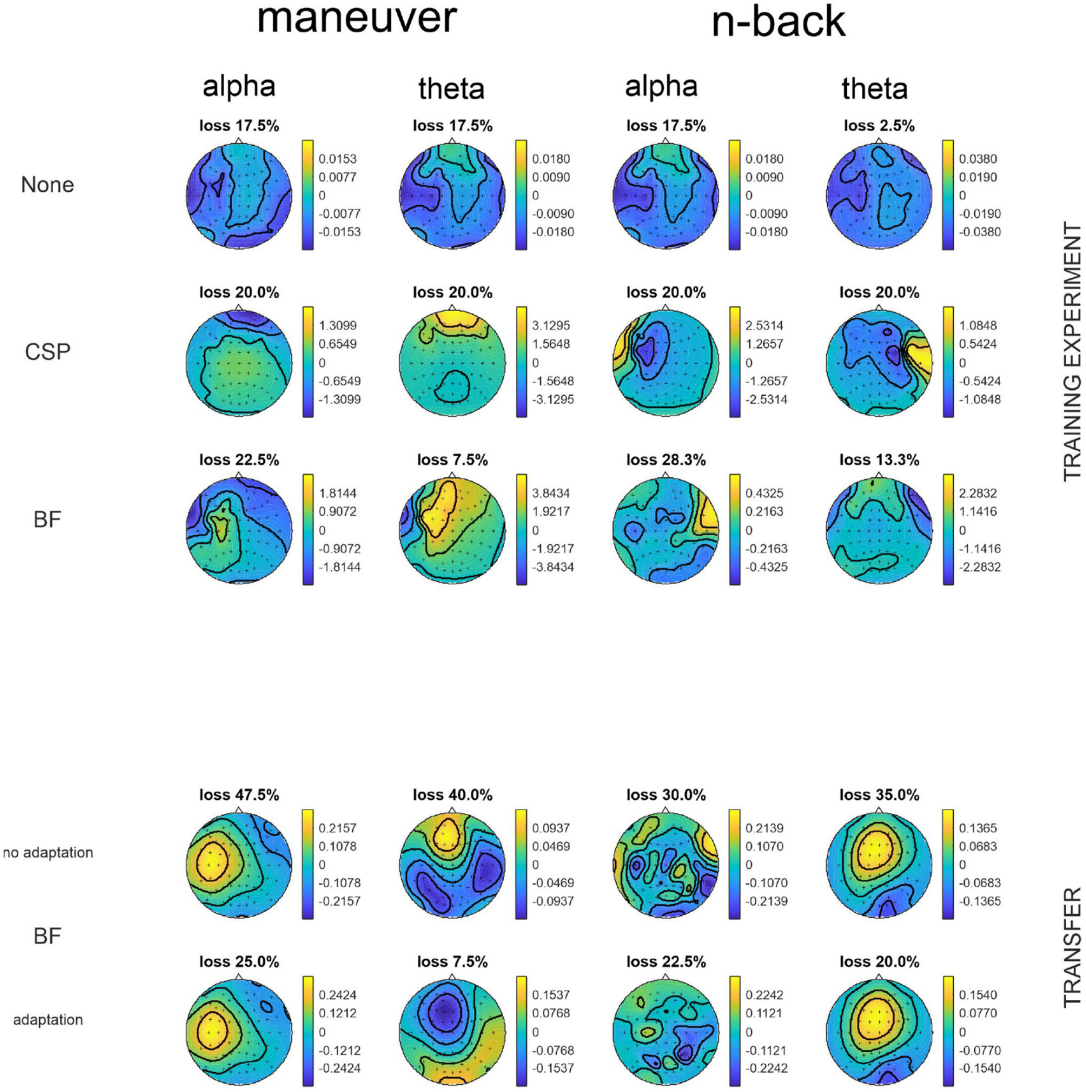
The most discriminative patterns in the different frequency bands for exemplary subject 5. On top the data was cross-validated while on the bottom the respective transfer classes were trained on the other task and then applied with or without adaptation. For no filtering, the LDA classifier pattern is shown with the overall normalized loss, while in the other cases the spatial patterns with the lowest normalized loss with the corresponding normalized loss in single component classification. While No Filtering lead to the classifier picking a combination of different sources (brain, eyes, and muscles), CSP mainly sticked to eye artifacts for the maneuvering while in the *n-back* the resulting patters could originate from yaw muscles or temporal cortices. Small changes in the patterns lead to lower losses for the adapted BF. The non-adapted patterns stay similar to their original patterns, as the covariances do not change much (not shown). Here, mainly broad activation lead to the lowest loss. Adaptation can also drastically change the pattern (see alpha in *n-back*) but mostly stays very similar (the flip in sign is meaningless).

The patterns of most discriminative components measured by single feature cross-validation within conditions contained a lot of patterns that can be identified as most probably stemming from eyes and muscles, as can be observed in Figure 2 on the top. This was stronger for the maneuvering task, while also found in the more controlled *n-back* task. Some patterns in the *n-back* task could actually display either yaw muscle activity but could also be related to temporal lobe originated oscillations. In the transfer between conditions, the lowest errors were elicited by different patterns that can be assumed to be mainly stemming from larger neural activation patterns in more central areas.

## 4. Conclusions

Filter adaptation to new settings lead to the ability to transfer classifiers between different experimental conditions for both CSP and beamforming while within condition no spatial filtering delivered the least errors. For CSP the adaptation was working only in one direction, while for beamforming it worked in both directions. The normalized losses were around 35% in the other conditions which is acceptable respecting the very different induction of workload using either a maneuvering or an auditory secondary task. This could imply extraction of generally meaningful components related to cognitive workload as they transfer between different settings. The other classifications seem over-fit to the experimental paradigm, as well as arbitrary statistics of each condition. In generalizability, BF with adaptation is the tool of choice.

The patterns are still not perfectly separable between neural and other origin, and a further step to improve this would be to use actual regions of interest (ROI) for the beamforming approach. We decided for an approach using all sources of the head model as a region of interest for the algorithm to find out correlated patterns separable from the noise out of the data. Putting more prior knowledge in the form of expected sources as ROIs into the model could improve results but could also lead to actually not detecting the meaningful information. This has to be further investigated in another study with narrowed ROIs. Problematic is, e.g., that the auditory nature of the *n-back* makes classical occipital alpha as is commonly related to cognitive workload often not a straightforward property because it is rather linked to visual attention, not auditory.

The eye- and muscle-based components in the patterns could be related to individual strategies, as well as systematic movements in certain maneuvering conditions. Patterns that can be found by CSP can in general mean two things—artifacts in single trials that occurred randomly more often in one class (Blankertz et al., 2008), or discriminative artifacts that differ systematically between both classes. As in the *n-back* condition the control of the ship was exactly identically, while also the *n-back* task did not differ in behavior only in the load of the memory task, the interpretation that individual (probably automatic) strategies in remembering the numbers was the only discriminative factor is reasonable. In the maneuvering task, it is more likely that certain viewing angles specific to different phases (next to the ship vs. looking straight at the bow) and steering movements make up most of the discriminative artifacts. But also here individual strategies or movement patterns related to high workload could play their part. In a preliminary analysis, we have compared the patterns between high and low workload and could see some patterns most likely related to neck muscle and eye artifacts. Also participant observations show a clearly different body pose and look direction in the different levels. This has to be further investigated.

As these systematic artifacts (in the sense of classical EEG interpretation) are highly specific to each of the conditions, they do not transfer well. This is underlined by the fact that within conditions the eye and muscle components lead to the lowest normalized loss, while in the transfer components stemming most likely from neural origin have the lowest normalized losses.

On the other hand, preliminary investigations of patterns and related filters revealed that the filters from one condition applied to the other condition extract similar patterns which do not lead to satisfactory normalized losses. Adapting the filters only slightly changed those patterns and permitted a normalized loss level significantly different from chance level. This could actually mean that it is not necessarily only the general artifact level that changes but also the non-stationarity of the neural signals themselves.

One open question that is contrary to common findings in controlled laboratory experiments is the high performance of the no spatial filtering approach within conditions. There is almost no transfer to other settings for no spatial filtering, so the classifiers seem rather condition specific. This could be caused by systematic contributions from eyes and muscles. As their sources lie in the scalp surface in vicinity to the electrodes they are less affected by spatial smearing due to volume conduction in the head than currents from neural sources which have to pass the low-conductive skull on their way. This makes them more local and spatial filtering that usually helps drastically with brain-only sources as in highly controlled experiments is not needed here for source separation. Looking at the patterns in our results revealed that indeed mainly channels on the circumference of the EEG cap have the largest absolute values.

Limitations of this study are in the adaptation of the spatial filters, as they are recalculated after every single trial. This could in general lead to different patterns and filters after a certain time. The classification is, however, not adapted and expects similar feature distributions which can decrease performance. Nevertheless, in our setting, a reasonable transferability with this approach has been reached. A better adaptation of the spatial filters, as well as a not yet investigated additional adaptation of the classifier could thus further improve results.

An actual source localization could shed more light onto the signals' origin and is planned for the near future when our head model that involves muscle and eye sources is finished.

No obvious difference could be found between the subject that had only 32 electrodes recorded and the others. This gives a hint toward that less electrodes could be sufficient for the analysis but has to be further investigated.

## Data Availability Statement

The original contributions presented in the study are included in the article/supplementary material, further inquiries can be directed to the corresponding author.

## Ethics Statement

The studies involving human participants were reviewed and approved by Philips research Ethics Committee, Eindhoven, The Netherlands. The patients/participants provided their written informed consent to participate in this study.

## Author Contributions

DM and BB planned and conducted the original study together with MARIN, The Netherlands and K+S Projects. DM had the main idea for this study and conducted the analysis in close corporation with BB. DM crafted the first draft of the article, which BB and DM finalized together. All authors contributed to the article and approved the submitted version.

## Funding

This original study was mainly funded by MARIN, The Netherlands. The research leading to these results has also received funding from the European Union Seventh Framework Programme (FP7/2007-2013) under grant agreement no 611570 (MindSee), and by the BMBF (contract 01GQ0850, BFNT).

## Conflict of Interest

The authors declare that the research was conducted in the absence of any commercial or financial relationships that could be construed as a potential conflict of interest.

## Publisher's Note

All claims expressed in this article are solely those of the authors and do not necessarily represent those of their affiliated organizations, or those of the publisher, the editors and the reviewers. Any product that may be evaluated in this article, or claim that may be made by its manufacturer, is not guaranteed or endorsed by the publisher.
